# Genomic Ancestry, Self-Reported “Color” and Quantitative Measures of Skin Pigmentation in Brazilian Admixed Siblings

**DOI:** 10.1371/journal.pone.0027162

**Published:** 2011-11-02

**Authors:** Tailce K. M. Leite, Rômulo M. C. Fonseca, Nanci M. de França, Esteban J. Parra, Rinaldo W. Pereira

**Affiliations:** 1 Programa de Pós-Graduação em Educação Física, Universidade Católica de Brasília, Brasília, Distrito Federal, Brazil; 2 Department of Anthropology, University of Toronto at Mississauga, Mississauga, Ontario, Canada; 3 Programa de Pós-Graduação em Ciências Genômicas e Biotecnologia, Universidade Católica de Brasília, Brasília, Distrito Federal, Brazil; University of Bristol, United Kingdom

## Abstract

A current concern in genetic epidemiology studies in admixed populations is that population stratification can lead to spurious results. The Brazilian census classifies individuals according to self-reported “color”, but several studies have demonstrated that stratifying according to “color” is not a useful strategy to control for population structure, due to the dissociation between self-reported “color” and genomic ancestry. We report the results of a study in a group of Brazilian siblings in which we measured skin pigmentation using a reflectometer, and estimated genomic ancestry using 21 Ancestry Informative Markers (AIMs). Self-reported “color”, according to the Brazilian census, was also available for each participant. This made it possible to evaluate the relationship between self-reported “color” and skin pigmentation, self-reported “color” and genomic ancestry, and skin pigmentation and genomic ancestry. We observed that, although there were significant differences between the three “color” groups in genomic ancestry and skin pigmentation, there was considerable dispersion within each group and substantial overlap between groups. We also saw that there was no good agreement between the “color” categories reported by each member of the sibling pair: 30 out of 86 sibling pairs reported different “color”, and in some cases, the sibling reporting the darker “color” category had lighter skin pigmentation. Socioeconomic status was significantly associated with self-reported “color” and genomic ancestry in this sample. This and other studies show that subjective classifications based on self-reported “color”, such as the one that is used in the Brazilian census, are inadequate to describe the population structure present in recently admixed populations. Finally, we observed that one of the AIMs included in the panel (rs1426654), which is located in the known pigmentation gene *SLC24A5*, was strongly associated with skin pigmentation in this sample.

## Introduction

Population structure can be a source of confounding in genetic epidemiology studies. Typically, the effect of population stratification is to inflate the rate of false positives in case-control or quantitative trait studies. This is particularly the case in populations that are the result of recent admixture between continental groups [1]. The Brazilian population is primarily the result of admixture between European, African and Native American groups [2]–[5]. The Brazilian census classifies individuals according to “color” or “race”, and includes the following categories: “Branca” (i.e. “white”), “Parda” (i.e. “brown”), “Preta” (i.e. “black”), “Amarela” (i.e. “yellow”) and “Indígena” (i.e. “indigenous”). However, several studies have demonstrated that stratifying according to “color” is not a useful strategy to control for population structure, due to the dissociation between self-reported “color” and genomic ancestry [6]–[11]. The best strategy to identify, and control for, population stratification in association studies in recently admixed groups is to use AIMs, which are genetic markers showing large frequency differences between the parental populations, to estimate individual ancestry proportions and to include them as covariates in the statistical analysis [1], [12].

Although there have been several studies in Brazil exploring the relationship between “color” (either self-reported or reported by observers) and genomic ancestry estimated with AIMs [6]–[11], they have not incorporated quantitative measures of skin pigmentation obtained with reflectometry [13], [14]. In this manuscript, we report the results of a study in a group of Brazilian siblings in which we measured constitutive skin pigmentation (i.e. pigmentation in unexposed areas of the skin) using a reflectometer, and estimated genomic ancestry using 21 AIMs. Self-reported “color”, according to the Brazilian census (“white”, “brown” or “black”), was also available for each participant. This made it possible to evaluate the relationship between self-reported “color” and skin pigmentation (expressed quantitatively as melanin index), self-reported “color” and genomic ancestry, and skin pigmentation and genomic ancestry. We observed that, although there were significant differences between the three “color” groups in genomic ancestry and skin pigmentation, there was considerable dispersion within each group and substantial overlap between groups. We also saw that in many instances, each member of the sibling pair reported different “color”, although on average there were no significant differences between siblings in melanin index. These results emphasize the complex relationship between self-reported “color”, skin pigmentation and genomic ancestry. Finally, we observed that one of the markers included in the AIMs panel (rs1426654), which is located in the known pigmentation gene *SLC24A5*, had a strong association with melanin index in this sample.

## Methods

The study was approved by the Research Ethics Committee in the Catholic University of Brasília (CEP/UCB N° 078/2006) and written informed consent was obtained from each volunteer or responsible in cases of participants with less than 18 years of age.

The sample comprised 86 pairs of healthy female full siblings (n = 172), previously confirmed by short tandem repeat genotyping [15], with a mean age of 15.60±2.19 years, which were recruited from public high schools in Brasília, Brazil. “Color” was self-reported according to the Brazilian official census categories [16]: “White”, “Brown”, “Black”, “Yellow” and “Indigenous”. None of the participants checked the categories “Yellow” or “Indigenous”, so the study only includes three “color” categories. Socioeconomic status (SES) was evaluated using the Brazilian Criteria of Economic Classification [17], which is based on an evaluation of a household's assets, as well as education levels. SES is reported in five categories: A, B, C, D and E, in decreasing order of SES.

Skin pigmentation was measured using a DermaSpectrometer (Cortex Technology, Hadsund, Denmark). The Dermaspectrometer estimates the amount of melanin in the skin from the amount of light reflected back to the machine in the red and green wavelengths of the light spectrum. The amount of melanin is expressed as the “Melanin Index”, which typically ranges from the low 20 s to almost 100 s, with individuals with the lightest skin pigmentation having the lowest values and those with the darkest pigmentation having the highest [18]. Skin pigmentation was measured in triplicate for both arms, near the armpit, and the values were averaged to obtain an estimate of melanin index.

To estimate genomic ancestry, 21 AIMs were genotyped, after DNA extraction from blood leucocytes using the salting out method [19]. Genotyping encompassed a multiplex PCR (Qiagen^®^ Multiplex PCR Kit), followed by purification with *exonuclease-I* and *shrimp alkaline phosphatase*, and a single base extension (ABI Prism^®^ SNaPshot^®^ Multiplex Kit, Applied Biosystems, Foster City, CA, USA) followed by purification with *shrimp alkaline phosphatase*. After capillary electrophoresis (ABI 3130×l Applied Biosystems), the genotypes were scored with the GeneMapper^TM^ 4.0 software (Applied Biosystems, Foster City, CA, USA). Information about the allele frequencies of each AIM in the three parental populations, as well as estimates of ancestry information content, expressed as f-value [20] and the informativeness statistic, In, [21] are reported in Table 1. Samples from Botswana, Cameroon, Ghana, and Senegal (AFR n = 120), European–Americans from Baltimore and Chicago (EUR n = 78) and Zapotec from Mexico (AMR n = ) were used as representative of the African, European and Native American parental populations, respectively.

**Table 1:** Allele frequency of ancestry markers in parental population and Informativeness (In).

					AFR/EUR		AFR/AMR		AMR/EUR	
rs #	Allele 1	EUR	AFR	AMR	f	In	f	In	f	In
rs1240709	A	0,766	0,050	0,103	0,530	0,304	0,010	0,005	0,447	0,246
rs2065160	C	0,088	0,487	0,875	0,195	0,105	0,173	0,091	0,620	0,355
rs2814778	G	0,006	0,983	0,019	0,953	0,630	0,930	0,604	0,003	0,002
rs3796384	C	0,154	0,783	0,875	0,398	0,215	0,015	0,007	0,520	0,290
rs2278354	G	0,120	0,703	0,839	0,351	0,190	0,026	0,013	0,518	0,288
rs267071	C	0,654	0,088	1,000	0,343	0,188	0,838	0,540	0,209	0,138
rs222541	G	0,257	0,971	0,018	0,538	0,316	0,908	0,582	0,120	0,070
rs1480642	C	0,994	0,121	0,621	0,772	0,483	0,268	0,143	0,223	0,139
rs4305737	A	0,250	0,921	1,000	0,464	0,259	0,041	0,028	0,600	0,380
rs285	T	0,578	0,954	0,448	0,197	0,111	0,305	0,173	0,017	0,008
rs3176921	T	0,929	0,238	0,983	0,493	0,278	0,584	0,351	0,017	0,009
rs803733	C	0,880	0,013	0,411	0,761	0,470	0,237	0,144	0,241	0,128
rs7349	T	0,062	0,969	0,000	0,824	0,507	0,939	0,623	0,032	0,022
rs730570	A	0,896	0,197	0,054	0,492	0,274	0,047	0,025	0,712	0,421
rs1129038	C	0,224	0,996	0,983	0,625	0,389	0,004	0,002	0,601	0,362
rs1426654	G	0,013	0,967	0,931	0,910	0,586	0,007	0,003	0,846	0,532
rs734780	C	0,062	0,710	0,854	0,444	0,250	0,030	0,015	0,633	0,366
rs730086	C	0,667	0,063	1,000	0,393	0,220	0,881	0,574	0,200	0,132
rs6034866	A	0,083	0,954	0,143	0,759	0,456	0,664	0,390	0,009	0,004
rs310612	A	0,763	0,013	0,966	0,593	0,360	0,909	0,584	0,088	0,048
rs727563	C	0,253	0,811	0,948	0,312	0,166	0,045	0,024	0,504	0,288

rs # = reference sequence number; EUR = European; AFR = African; AMR = Amerindian; f = f−value; In = Informativeness statistic.

Individual ancestry was calculated with the program ADMIXMAP (http://homepages.ed.ac.uk/pmckeigu/admixmap/) using 2,500 iterations for the burnin period and 10,000 iterations to measure parameter data. This program also provides an estimate of the sum of intensities parameter, which is equivalent to the average number of generations since admixture. The individual ancestry estimates obtained with ADMIXMAP are highly correlated to the estimates obtained with the program STRUCTURE 2.3.3 [22]: AMR Rho = 0.970, p<0.001; AFR Rho = 0.987, p<0.001; EUR Rho = 0.980, p<0.001. We provide as supplementary information the individual ancestry estimates obtained with ADMIXMAP and STRUCTURE, as well as the confidence intervals of the individual ancestry estimates from STRUCTURE (Table S1). ADMIXMAP was also used to evaluate the association of the genetic markers with melanin index, after conditioning for individual ancestry. The p−values were adjusted using the Bonferroni correction for multiple comparisons.

Bland-Altman plots [23] were used to evaluate the agreement of genomic ancestry and melanin index between siblings. For the categorical variable “color”, a Cohen's Kappa analysis was carried out for the same purpose [24]. The relationship of “color” and melanin index, “color” and genomic ancestry, SES and melanin index and SES and genomic ancestry was evaluated using ANOVA, because there were no major departures from the assumptions required by this test. The use of the non-parametric Kruskal-Wallis test gave almost identical results. In order to explore the relationship of melanin index and genomic ancestry in the total sample, we used Spearman's nonparametric correlation and the relationship of “color” and SES was evaluated using a chi-square test. These statistical analyses were carried out with the software SPSS 16.0, using 0.05 as the significance level. As our study deals with siblings, some of these analyses were also carried out in unrelated subjects, and the results were overly similar to those observed for the whole group.

## Results

Table 2 shows the main characteristics of the sample. The average age was 15.6 years, the average melanin index was 36.5, and the average European, West African and Native American contributions were 69%, 21% and 10%, respectively. For the variable “color”, 31.4% of the participants reported to be “white”, 60.5% reported to be “brown” and 8.1% reported to be “black”.

**Table 2:** Main characteristics of the sample.

		Total Group (n = 172)	
Age (years)			15.60±2.19
Pigmentation (Melanin Index)			36.50±3.46
European Ancestry			0.69±0.08
African Ancestry			0.21±0.07
Amerindian Ancestry			0.10±0.03
Sum of intensities			8.8
	White		54 (31.4%)
“Color”	Brown		104 (60.5%)
	Black		14 (8.1%)

Data presented as mean ± standard deviation or frequencies.

### Genomic ancestry, melanin index and “color” in pairs of siblings

**Figure 1:**
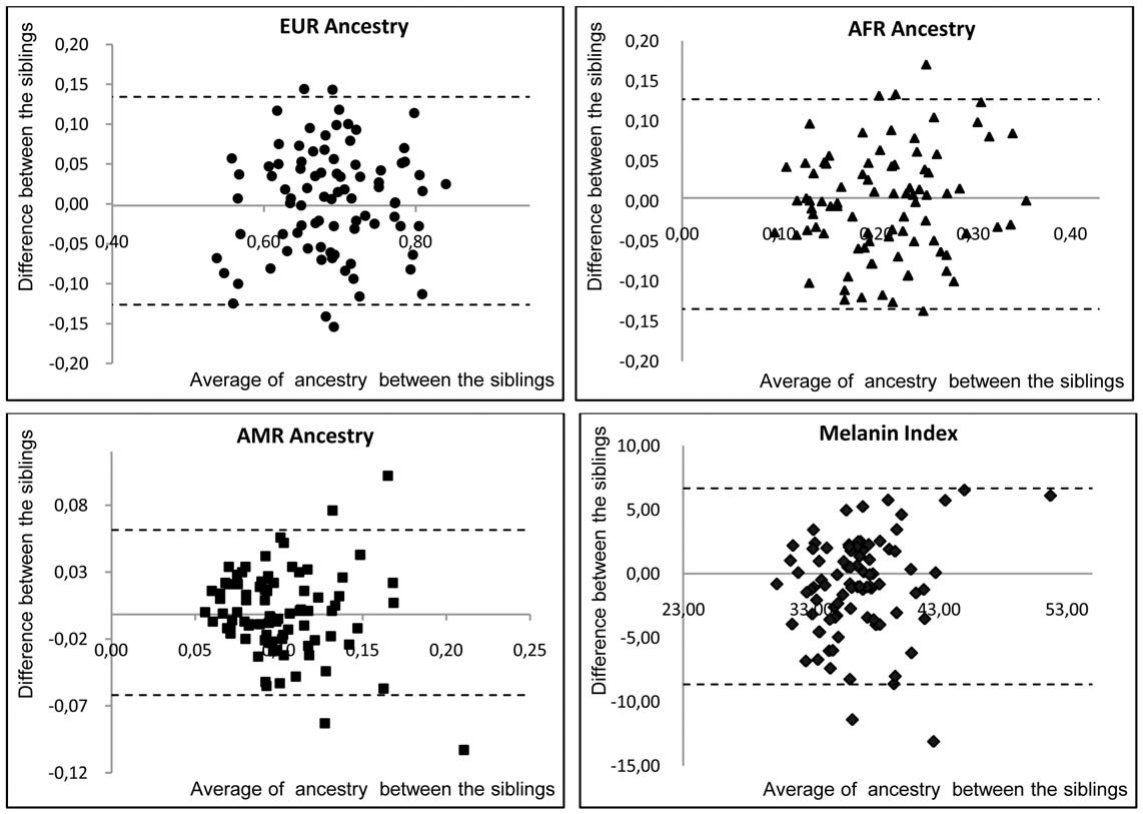
Bland-Altman Analysis.

Figure 1 depicts Bland-Altman plots showing the agreement of the genomic ancestry proportions and melanin index values between siblings. Overall, there is good agreement between siblings, with very few pairs differing by more than 15% (Figure 1).

**Figure 2:**
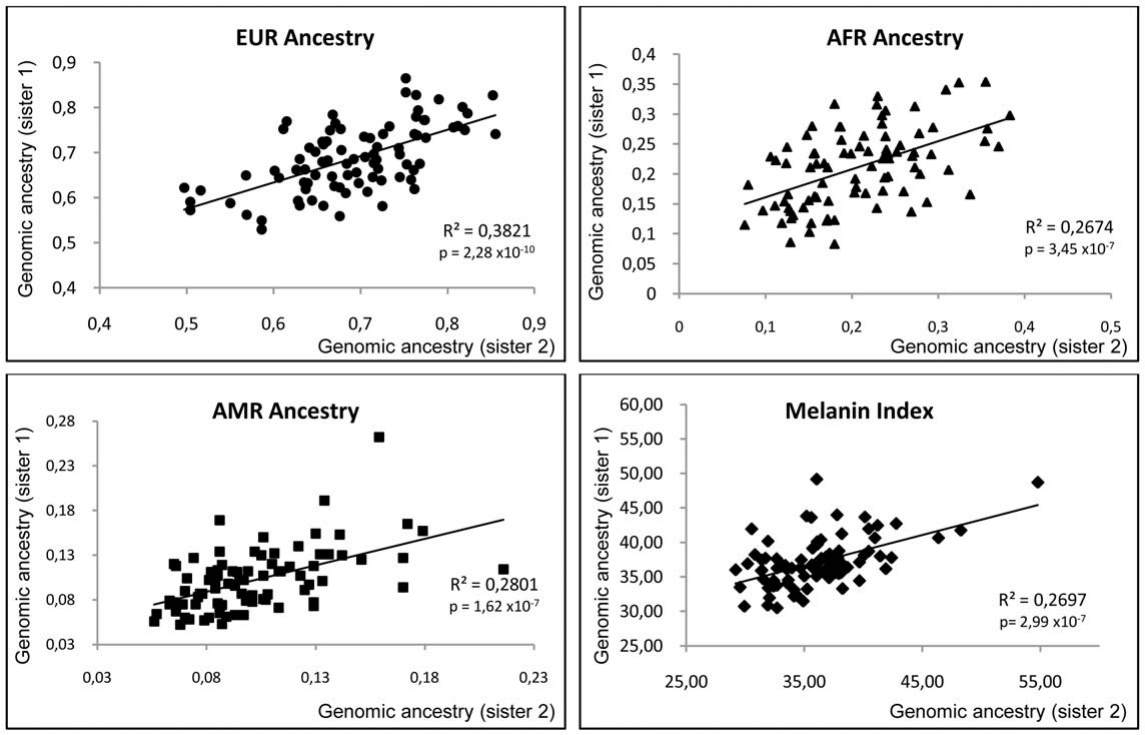
Scatter plot from ancestry proportions and pigmentation between siblings.

Another way to present the data is by means of scatter plots, which are depicted in Figure 2. As expected, there is a significant correlation of ancestry proportions and melanin index values between siblings, with coefficients of determination ranging from 0.26 to 0.38 (Figure 2).

Table 3 is a contingency table depicting the “color” categories reported by each pair of siblings. The agreement is described by Cohen's Kappa coefficient. Under a perfect agreement, the Kappa coefficient would be 1. Of the 86 comparisons, 30 were discordant, and the Kappa coefficient for the total sample was 0.342.

**Table 3:** Contingency table of reported “color”.

		Sibling 1						
		White	Brown	Black	Total			
							Kappa	p
	White	16	10	0	26		**0.407**	**<0.001**
Sibling 2	Brown	12	37	5	54		**0.272**	**0.011**
	Black	0	3	3	6		**0.379**	**<0.001**
	Total	28	50	8	86		**0.342**	**<0.001**

### Genomic ancestry and “color”

**Figure 3:**
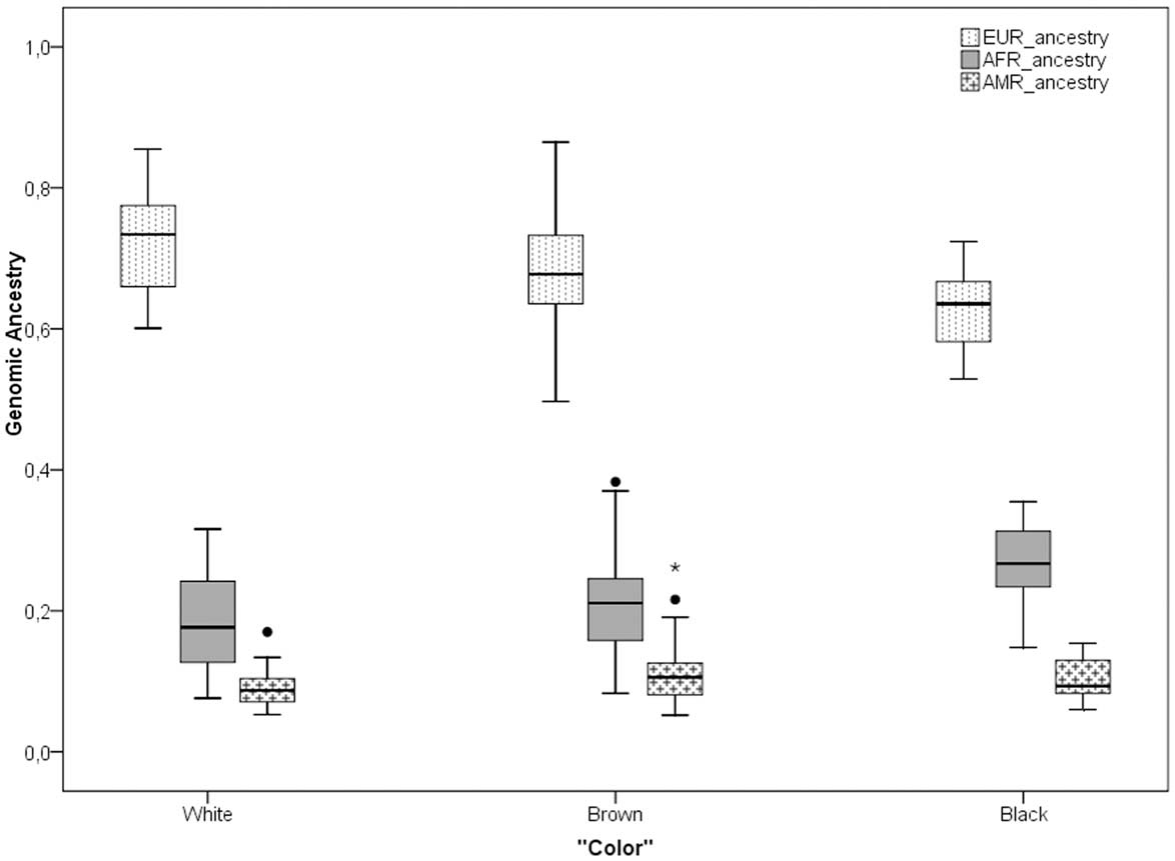
Genetic ancestry proportions×“Color”.

Table 4 shows the average ancestral proportions estimated with the panel of AIMs in each of the “color” categories reported by the participants. The average European ancestry in the “white”, “brown” and “black” groups was estimated as 72%, 68% and 63%, respectively. Conversely, the average African ancestry in the “white”, “brown” and “black” groups were 19%, 21% and 27%, respectively. The Native American ancestry ranged from 9 to 11%. The differences in ancestry proportions between “color” groups are statistically significant. However, a box plot showing the distribution of genomic ancestry in each “color” category indicates that there is substantial dispersion within and considerable overlap between “color” categories (Figure 3).

**Table 4:** Genetic ancestry× “Color” (ANOVA with Bonferroni correction).

	White (n = 54)	Brown (n = 104)	Black (n = 14)	p
European Ancestry	0.72±0.07+	0.68±0.07	0.63±0.06++	0.001
African Ancestry	0.19±0.07*	0.21±0.07	0.27±0.06**	0.001
Amerindian Ancestry	0.09±0.02#	0.11±0.04	0.10±0.03	0.006

Data presented as mean ± standard deviation.

+Different from Brown and Black;

++Different from White;

*Different from Black;

**Different from White and Brown;

#Different from Brown.

### Melanin index and “color”

**Figure 4:**
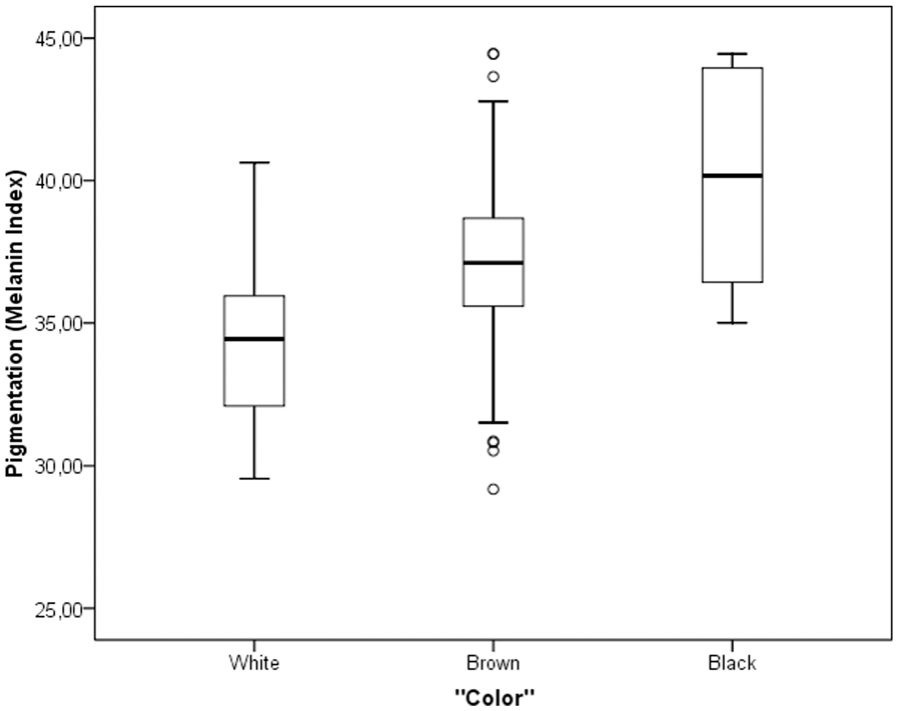
Melanin Index and “Color”.

Table 5 reports the average melanin index in each of the three “color” categories. The average melanin indices in the “white”, “brown” and “black” categories were 34.25, 37.17 and 40.26, respectively, and these differences are statistically significant. Similarly to what was observed for genomic ancestry, there is also considerable variation within, and overlap between “color” groups in skin pigmentation measured quantitatively (Figure 4).

**Table 5:** Skin pigmentation× “Color” (ANOVA with Bonferroni correction).

	White (n = 54)	Brown (n = 104)	Black (n = 14)	p
Melanin Index	34.25±2.56‡	37.17±3.11‡	40.26±3.80‡	0.001

Data presented as mean ± standard deviation.

‡ All groups with statistical significant differences.

### Genomic ancestry and melanin index

The analysis of the relationship of genomic ancestry and melanin index using Spearman's rank order correlation indicated that African ancestry was positively correlated with melanin index (rho = 0.258, p = 0.001), and European ancestry was inversely correlated with melanin index (rho = −0.291, p<0.001). No significant correlation was observed between Native American ancestry and melanin index (rho = 0.130, p = 0.088).

### Socioeconomic status, self-reported “color”, skin pigmentation and genomic ancestry

Table 6 and table 7 show the relationship of SES and self-reported “color”, SES and genomic ancestry and SES and skin pigmentation. Using a chi-square test, we observed that SES and “color” are not independent variables: individuals who self-reported to be “white” tend to have higher SES than individuals reporting to be “black” (X^2^ = 19.38, p = 0.004, Table 6). Similarly, the ANOVA analysis indicated that individuals with higher European ancestry tend to have higher SES (p = 0.002, Table 7). No significant association was observed between skin pigmentation and SES (Table 7).

**Table 6:** “Color” ×SES (Chi-square test).

	Socioeconomic Status						
		A	B	C	D	X^2^	p
**“White”**	Ob	3	32	18	1		
	Exp	1.3	23.9	23.9	5.0		
**“Brown”**	Ob	1	39	53	11		
	Exp	2.4	46.0	46.0	9.7		
**“Black”**	Ob	0	5	5	4		
	Exp	0.3	6.2	6.2	1.3		
**Total**		4	76	76	16	19.38	**0.004**

SES = socioeconomic status; Ob = Observed values; Exp = expected values; X^2^ = chi-square value.

**Table 7:** Genetic ancestry and Skin Pigmentation×SES (ANOVA with Bonferroni correction).

	A (n = 4)	B (n = 76)	C (n = 76)	D ( = 16)	p
European Ancestry	0.70±0.02	0.71±0.08*	0.67±0.07	0.65±0.07	**0.002**
African Ancestry	0.21±0.03	0.19±0.07++	0.22±0.07	0.24±0.07	**0.021**
Amerindian Ancestry	0.09±0.03	0.10±0.03	0.11±0.03	0.11±0.03	0.068
Pigmentation	35.64±4.98	36.13±3.38	36.74±3.44	37.33±3.63	0.498

SES = socioeconomic status; Data presented as mean ± standard deviation.

*Different from C and D;

++Different from D.

### Association of AIMs with melanin index

Table 8 shows the results of the tests of association of each AIM with melanin index, conditioning for individual ancestry, which were carried out with the program ADMIXMAP. The only significant marker after correction for multiple testing was rs1426654 (p−value = 4.2×10^−12^). This marker is located within the pigmentation gene *SLC24A5*, which has been associated with skin pigmentation in numerous studies [25]–[31]. An unconstrained regression analysis including the rs1426654 genotypes as a fixed factor and individual ancestry as a covariate showed that AG heterozygotes are associated with an increase of 2.17 melanin index units (p<0,001), and GG homozygotes with an increase of 4.96 melanin index units (p<0,001), compared with homozygotes for the derived A allele.

**Table 8:** Allelic Association Test (Ancestry×Pigmentation).

Locus	Chromosome	cM	pValue
rs1240709	1	2.24	0.482
rs2065160	1	164.82	0.088
rs2814778	1	222.38	0.675
rs3796384	2	90.59	0.101
rs2278354	3	26.90	0.895
rs267071	3	134.26	0.227
rs222541	4	117.26	0.637
rs1480642	4	154.06	0.586
rs4305737	4	164.39	0.012
rs285	5	48.70	0.310
rs3176921	5	93.94	0.257
rs803733	6	163.13	0.571
rs7349	7	60.37	0.973
rs730570	8	118.49	0.142
rs1129038	9	14.76	0.081
rs1426654*	9	43.57	4.28E−12
rs734780	9	97.05	0.270
rs730086	10	84.51	0.348
rs6034866	11	43.27	0.972
rs310612	11	108.50	0.919
rs727563	12	54.03	0.313

*Significant level after Bonferroni correction: p ≤0,002.

## Discussion

In this manuscript, we studied the relationship between self-reported “color”, quantitative measures of skin pigmentation, and genomic ancestry in a sample of full siblings from Brasilia. With respect to previous research in Brazil, this study is novel in that we measured skin pigmentation objectively using reflectometry and we sampled full siblings, which allowed us to evaluate the agreement of self-reported “color” and melanin index in each pair of siblings. Using a panel of 21 AIMs, we estimated that this sample has a European contribution of 69%, an African contribution of 21% and a Native American contribution of 10% (Table 2). These relative contributions are quite similar to those that have been reported in previous studies using AIMs in Brazil. For example, Lins et al. [4], estimated parental contributions in samples from different geopolitical regions in Brazil, and reported that European ancestry ranged from 69% to 88%, African ancestry from 7% to 19%, and Native American ancestry from 5% to 12%. Pena et al. [8] evaluated genetic ancestry in North, Northeast, Southeast and South regions of Brazil and reported similar figures, with the European contribution ranging from 60.1% to 79.5%, the African contribution from 10.3% to 29.3% and the Native American contributions from 7.3% to 18.55%. Using the program ADMIXMAP, we also obtained an estimate of the number of generations since admixture, which was approximately 9 generations, approximately 270 years ago, assuming 30 years per generation according to Helgason [32], which is quite consistent with historical data. However, it is important to mention that this estimate is based on a limited number of AIMs, so it has to be interpreted with caution.

As expected, we observed that the ancestral proportions estimated for each sibling were significantly correlated (Figure 2). For most pairs of siblings, the differences between the estimates were lower than 10%, and very few estimates differed by more than 15% (Figure 1). It is important to note that using 21 AIMs makes it possible to obtain a precise estimate of ancestral proportions in the sample, but the number of AIMs is not high enough to obtain precise estimates at the individual level. Therefore, it is not surprising to observe some dispersion in the individual admixture proportions estimated for the pairs of siblings. We also saw that there is a significant correlation of the melanin index values of the sibling pairs (R^2^ = 0.27, p−value = 2.99×10^−7^).

One interesting finding of this study is that there was no good agreement between the “color” categories reported by each member of the sibling pair. Under a perfect agreement, we would expect that both members of each pair would report the same “color” category: “white”, “brown” or “black”, and this would have been reflected in a Kappa coefficient of 1. However, in 30 out of the 86 sibling pairs (35%), each sibling reported a different “color” category (white/brown: 22 pairs, or black/brown: 8 pairs, Table 3). The value of kappa for the total sample was 0.34. There is no universal consensus about the interpretation of the Kappa values, but most statisticians would consider that for a good level of agreement, kappa should be higher than 0.61 [33]. Although the data clearly indicates that the agreement is not good, it is difficult to speculate about the reasons for these discrepancies. However, given that we have objective measures of skin pigmentation obtained with reflectometry, we can explore if the discrepant responses that the siblings provide for “color”, can be explained by actual skin pigmentation differences between siblings. One way to do this is to estimate an absolute delta value measuring the difference in pigmentation between siblings, and test if those pairs of siblings reporting different “color” categories have significantly higher delta values (e.g. larger pigmentation differences) than those reporting the same “color” categories. This analysis showed that the pairs of siblings reporting different “color” categories had an average delta of 3.48 (±2.94) melanin units, whereas the pairs of siblings reporting the same “color” categories had a slightly lower average delta of 2.71 (±2.36) melanin units. Statistically, the differences between groups were not significant (t = 1.33, p = 0.19), indicating that actual differences in skin pigmentation are not the major reason for the lack of agreement between self-reported “color” between siblings. Also, in 7 out of 30 pairs of siblings reporting different “color” categories (23%), the sibling who reported the darker “color” category had a lower melanin index (e.g. lighter skin) than the other sibling, suggesting again that self-reported “color” is a poor proxy of melanin levels. There seem to be other factors, including sociocultural factors, involved in the substantial proportion of sibling pairs reporting discrepant “color” categories. We did not observe significant differences in SES between pairs of siblings reporting similar versus discrepant “color” categories (X^2^ = 1.17, p = 0.761). Similarly to what we have observed in our study, Santos et al. [9] reported that, among a group of students that were asked to report their “color” on two different occasions (four months apart), approximately 20% of them reported a different classification in both visits.

Irrespective of the factors involved in the discrepancies in self-reported “color” between siblings, it seems clear that stratifying the sample according to self-reported “color” is not a useful strategy to control for stratification. This is evident when analyzing the relationship of “color” and genomic ancestry (Figure 3 and Table 4), and “color” and melanin index (Figure 4 and Table 5). When comparing the ancestry contributions in each of the three “color” categories, we observed that there were significant differences in the expected directions: Individuals reporting to be “white” had, on average, higher European contribution than individuals reporting to be “brown”, and the lowest European contribution was observed among those reporting to be “black” (Table 4). The opposite is observed for the African contribution. However, from the plots presented in Figure 3, it is quite clear that there is considerable dispersion within each group, and also substantial overlap between groups. In summary, there is evidence indicating that there is a sizable amount of population stratification within each group. Something very similar is observed in the analysis of the relationship of “color” and melanin index. Individuals reporting to be “white” tend to have lower melanin index values (e.g. lighter skin pigmentation) than individuals reporting to be “brown”, and the highest melanin index values are observed in the group of individuals reporting to be “black”. Again, the differences between groups are statistically significant, but there is a broad distribution of melanin values in each group, and considerable overlap between groups. The relationship of “color” and genomic ancestry was also tested in a sample including only unrelated subjects, and similar results were observed. The relationship that we observed between self-reported “color” and melanin index is overly similar to that reported by Gravlee et al. [34], who studied the relationship between ascribed “color” (“*blanco*”, “*trigueño” or “negro”)* and melanin index in Puerto Rico.

It is also of interest to analyze the relationship between genomic ancestry and melanin index. In our sample, African contribution was positively correlated with melanin index, e.g. darker skin (rho = 0,258, p = 0.001) and European contribution was negatively associated with melanin index (rho = −0,291, p<0.001). These significant correlations indicate that there is population stratification in the sample. In a previous study, Parra et al. [13] evaluated the relationship of genomic ancestry (estimated with 21–34 AIMs) and melanin index (using the same instrument employed in this study, the DermaSpectrometer) in four admixed samples (African Americans, African Caribbeans, Puerto Ricans and Mexicans) and also reported significant correlations of genomic ancestry and melanin index in each of these samples. However, the strength of the correlation was quite variable, ranging from rho = 0.633 in Puerto Rico to rho = 0.212 in Mexico, reflecting varying levels of populations stratification. The correlation observed in our study (rho = 0.258) is substantially lower than that observed in the Puerto Rican (rho = 0.633), African American (rho = 0.440) and African Caribbean (rho = 0.375) samples, indicating that this Brazilian sample shows less stratification than in the aforementioned samples. In a study including 147 Hispanics and 15 Native Americans from New Mexico, Klimentidis et al. [35] also found a significant, although moderate, association between constitutive pigmentation and Amerindian genetic ancestry (r = 0.29, p = 0.003).

The availability of data on SES in this sample made it possible to evaluate the relationship of SES and self-reported “color”, SES and genomic ancestry, and SES and skin pigmentation. No significant association was found between SES and skin pigmentation. However, there were significant associations between SES and self-reported “color” and SES and genomic ancestry (Table 6 and table 7, respectively). Participants reporting to be “white” tend to have higher SES than those reporting other “color” categories. We also observed that the average European ancestry proportion was higher in individuals with higher SES. Previous studies in other countries in Latin America have reported similar associations between SES and genomic ancestry [36], [37]. These findings have important implications for association studies focused on diseases in which socioeconomic factors play an important role. For these diseases, there will be differences in SES between the sample of cases and controls. Because SES is also associated with genomic ancestry, it would also be expected that ancestry proportions will be different in the case and control groups. In such situations, there will be an inflation of false positives in the tests of association, unless individual ancestry proportions are included in the statistical models in order to correct for the effects of population stratification. A clear example of this can be found in a recent genome-wide study of type 2 diabetes in a sample from Mexico City [38]. The significant correlation between socioeconomic status and genomic ancestry also emphasizes the need to be cautious when exploring the relationship between genomic ancestry and disease risk. Significant associations between genomic ancestry and disease risk are sometimes explained by population differences in disease risk due to genetic factors. However, in some situations, the association of genomic ancestry with disease risk may be mediated primarily by the effect of socioeconomic factors.

Finally, we evaluated the association of the AIMs genotyped in this study and melanin index. We observed that the SNP rs1426654 was strongly associated with skin pigmentation (p−value = 4.2×10^−12^). This SNP is a non-synonymous polymorphism located in the *SLC24A5* gene, in which the G allele encodes an Alanine amino acid and the A allele encodes a Threonine at position 111 of the protein [25]. The rs1426654 shows a very unusual allele frequency distribution in human populations, with the ancestral Alanine allele fixed in African, East Asian and Native American populations, and the derived Threonine allele almost fixed in European populations [39]. For this reason, this SNP was included in the panel of AIMs used in this study. This polymorphism has one of the strongest effects reported for skin pigmentation and it has been associated with pigmentary traits in numerous studies [25]–[31], so it is not surprising that we have observed a significant association of rs1426654 and melanin index in our sample. In agreement with previous reports, we estimated that homozygotes for the ancestral Alanine allele are associated with an increase of 4.96 melanin units and heterozygotes are associated with an increase of 2.17 melanin units, with respect to the homozygotes for the derived Threonine allele.

This study has a number of limitations. First, the sample consists of young female siblings recruited in the State of Brasilia, and may not be generalizable to other demographic groups or geographic regions of Brazil. Second, the sample size was relatively small, and therefore, the statistical power to identify small effects is limited. Finally, the ancestry estimates were based on 21 AIMs. Although the AIMs are highly informative, and the estimates of ancestral proportions in the sample is quite precise, the individual ancestry estimates are associated with relatively large confidence intervals (see supporting information, Table S1). However, simulation studies [40] and comparisons of admixture estimates based on more than 1,000 AIMs with those based on reduced numbers of AIMs [41], indicate that individual ancestry estimates based on 25–30 AIMs show relatively high correlations with the true ancestral proportions (r>0.75) [40] or the ancestry estimates based on a large number of AIMs (r = 0.89) [41].

In summary, we describe the relationship between self-reported “color” and skin pigmentation (expressed quantitatively as melanin index), self-reported “color” and genomic ancestry, and skin pigmentation and genomic ancestry in a sample of female siblings from Brasilia. This and other studies show that subjective classifications based on self-reported “color”, such as the one that is used in the Brazilian census, are inadequate to describe the population structure present in admixed populations. In association studies in recently admixed groups, the preferable approach to control for the effects of population stratification is to include individual ancestry proportions based on panels of AIMs as covariates in the statistical analysis.

## Supporting Information

Table S1 **Genomic Ancestry estimated by Admixmap and Structure.**
(XLSX)
